# Effect of Modulating Activity in the Right DLPFC on Revenge Behavior: Evidence From a Noninvasive Brain Stimulation Investigation

**DOI:** 10.3389/fpsyg.2020.608205

**Published:** 2021-02-01

**Authors:** Wanjun Zheng, Yuanping Tao, Yuzhen Li, Hang Ye, Jun Luo

**Affiliations:** ^1^Center for Economic Behavior and Decision-Making, Zhejiang University of Finance and Economics, Hangzhou, China; ^2^School of Economics, Zhejiang University of Finance and Economics, Hangzhou, China; ^3^Department of Radiology, The Guangxing Hospital Affiliated to Zhejiang Chinese Medical University, Hangzhou, China

**Keywords:** revenge behavior, selfishness, motivation, the dorsolateral prefrontal cortex, transcranial direct stimulation

## Abstract

Revenge is common in our daily lives, and people feel good when engaging in revenge behavior. However, revenge behavior is a complex process and remains somewhat of a puzzle of human behavior. Neuroimaging studies have revealed that revenge behaviors are associated with activation of a neural network containing the anterior cingulate cortex, ventral striatum, inferior frontal gyrus, and dorsolateral prefrontal cortex (DLPFC). Recent brain stimulation research using transcranial direct current stimulation (tDCS) and transcranial magnetic stimulation has shown a causal relationship between brain regions and revenge behaviors, but the findings have been mixed. In the present study, we aimed to study whether stimulation in the DLPFC can change participants’ revenge behavior in conditions where participants’ wealth was taken away in different ways. We adapted the moonlighting game and designed a new paradigm. Our study revealed that revenge behavior increased following activation in the right DLPFC, suggesting that the right DLPFC plays an important role in overriding self-interest and retaliation. In addition, our results revealed that the right DLPFC is crucial in revenge behavior related to the motivation of invasion.

## Introduction

Revenge mainly involves actions intended to harm someone after perceived harm to one’s well-being (Schumann and Ross, 2010; Elshout et al., 2015; Jackson et al., 2019). Despite its high costs and severe consequences, revenge is common in our daily lives, and people feel satisfied when engaging in revenge (Knutson, 2004; Gollwitzer et al., 2011; Chester and DeWall, 2016; Jackson et al., 2019). However, revenge behavior is a complex process that manifests in various patterns and remains somewhat of a puzzle of human behavior (Jackson et al., 2019). Hydraulic models of aggression have treated revenge as the result of a victim’s accumulation of negative energy after particular experiences (Freud, 1930; Miller, 1958), and clinical models of conflict considered revenge as the reverse side or objectionable alternative to forgiveness (Worthington and DiBlasio, 1990). Evolutionary theories have suggested that revenge is the best way for early humans to escape threats such as murder, theft and mate poaching (Sell et al., 2009; McCullough et al., 2013; Nowak et al., 2016).

Due to the complex causes of retaliation, revenge behaviors have also been researched in laboratory-based paradigms. In one of the first studies, Ford and Blegen discovered that people who took strong retaliatory measures faced less aggressive behavior (Ford and Blegen, 1992). Brüne found that most individuals responded in a tit-for-tat fashion in a scenario in which participants first play the part of the recipient in an ultimatum game (UG) and subsequently acted as a proposer in a dictator game (DG) played against opponents, as in the UG (Brüne et al., 2013). Abbink et al. (2000) introduced the moonlighting game in which a player can take money or pass money to another player who can either return money or punish the player. Abbink et al. (2000) found that revenge was much more compelling than reciprocity. Self-control studies have shown relationships between self-control and revenge behavior (Finkel and Campbell, 2001; Tangney et al., 2004; DeWall et al., 2010; Pronk et al., 2019). For example, Liu found that participants with lower self-control exhibited more revenge behavior when they were treated unfairly than those with high self-control (Liu and Li, 2020).

In accordance with behavioral studies examining revenge, recent neuroimaging studies have revealed that the decision-making process of revenge behavior is largely associated with the function of different brain regions. Conflict theory found that the impulsive desire for revenge is associated with neural activity in the anterior cingulate cortex (ACC), and the dorsolateral prefrontal cortex (DLPFC) receives signals for the implementation of cognitive control from the ACC in the revenge process (Kerns et al., 2004; Egner and Hirsch, 2005). Ricciardi et al. (2013) found that revenge reduction processes (i.e., forgiveness processes) were associated with significant covariations between the DLPFC, the ACC and the inferior frontal gyrus (IFG). Brüne reported that revenge behavior was accompanied by activation of the ventral striatum and that forgiveness behavior was correlated with activation of the right DLPFC (Brüne et al., 2013).

Although many brain regions, such as the ACC, IFG and ventral striatum, are important in the process of revenge behavior, the DLPFC, especially the right DLPFC, is thought to be the control area for the ACC and IFG (Clark, 2005; Maier et al., 2019). Although neuroimaging studies have allowed us to identify the associations between the DLPFC and revenge behavior (Buckholtz et al., 2008; Baumgartner et al., 2011; Strobel et al., 2011), the direct causal relationships remain unknown. Fortunately, brain stimulation technologies, such as transcranial direct current stimulation (tDCS), repetitive transcranial magnetic stimulation (rTMS) and continuous theta-burst stimulation (cTBS), create “virtual lesions” and provide a convenient method to identify causal relationships between revenge behavior and target brain regions. Maier et al. (2019) found that participants in the verum cTBS condition exhibited more revenge behavior than those in the placebo cTBS condition, which indicated that inhibition in the right DLPFC led to more revenge behavior. Similarly, Müller-Leinß et al. (2018) found that inhibiting the right DLPFC with repetitive transcranial magnetic stimulation induced increased retaliation towards previously unfair opponents. However, Knoch et al. (2006) found that disruption of the right DLPFC by rTMS decreased participants’ willingness to reject unfair offers, which suggested that participants were engaging in less revenge behavior. This finding also indicated that the function of the right DLPFC is to override basic human impulses related to self-interest by implementing culturally implemented fairness norms (Knoch et al., 2006). Consistent with Knoch’s study, Strang et al. (2015) found that dictators were less willing to punish while the right DLPFC was disrupted by rTMS compared with sham stimulation when they were imagining being in the role of recipient.

Obviously, the findings of associations between the DLPFC and revenge behavior are mixed and puzzling at the same time. The main reason may be that the studies above adopted different experimental paradigms. Maier et al. (2019) and Müller-Leinß et al. (2018) adopted the experimental design developed by Brüne et al. (2013). In their experiments, the participants first played a UG and subsequently played a DG in which the roles changed. Knoch et al. (2006) adopted the DG in which the receiver can deliver punishment by rejecting the offers when the proposal is unfair. Strang et al. (2015) adopted the DG and the dictator game with punishment option (DGp), which is similar to Knoch’s method. In the design of Brüne et al. (2013), participants can maximize their benefit by engaging in revenge behavior. However, in the design of Knoch et al. (2006), there is a trade-off between retaliation and benefit maximization. By and large, the revenge behaviors in the two kinds of experiments had the same results for the objects of revenge but had different results for those engaged in revenge.

Accordingly, to investigate revenge behavior and influencing factors, we adopted the adapted moonlight game (Abbink et al., 2000), which is partly similar to the DG with the punishment option. This behavioral measurement paradigm results in more realistic responses from participants when their money is taken away by other participants. In addition, the behavioral measure of retaliation across different tasks allowed us to judge whether participants who engaged in revenge behavior distinguished between losing tokens and anticipating the loss of tokens. Based on that, we examined the role of the right DLPFC in revenge behavior by using tDCS. We also investigated whether stimulation in the right DLPFC can change revenge behavior in conditions where tokens were not lost but the loss of tokens was anticipated. Finally, we discussed the revenge behavior in conditions where participants’ wealth was taken away and given to others by computer.

## Materials and Methods

### Subjects

We recruited a total of 184 healthy students (100 females; mean age of 20.36years) from Zhejiang University of Finance and Economics. The average age of females was 20.22, ranging from 18 to 25. The average age of males was 20.30, ranging from 18 to 27. Their majors are economics, finance, psychology, computer science, humanities, art, etc. All participants met the following criteria: right-handed; unfamiliar with tDCS; and no history of clinical impairments, psychiatric illness or neurological disorders. The participants were randomly assigned to either the role of someone who could take others’ tokens (*n*=92; 50 females) or the role of someone who could engage in revenge behavior (*n*=92; 50 females). The latter were randomly assigned to sham stimulation (*n*=32; 18 females), anodal tDCS (*n*=30; 16 females) or cathodal tDCS (*n*=30; 16 females) groups. The participants received a fixed show-up fee of 10 CNY (approximately 1.43US dollars) in addition to the money they gained during the experimental task. The entire experiment lasted approximately 60min; on average, participants received a payment of approximately 57.48 CNY (approximately 8.21US dollars) from the tasks, ranging from 0 to 106 CNY based on their performance and the computer program. The participants gave informed written consent before entering the study, which was approved by the Zhejiang University of Finance and Economics Ethics Committee. No participants reported any adverse side effects involving scalp pain or headaches.

### Transcranial Direct Current Stimulation

A weak direct current to the scalp was applied with tDCS *via* two saline-soaked surface sponge electrodes (35cm^2^). The current was constant and delivered by a battery-driven stimulator (a multichannel, noninvasive wireless tDCS neurostimulator; Starlab, Barcelona, Spain), which was controlled by a Bluetooth system. Generally, cathodal stimulation attenuates cortical excitability, whereas anodal stimulation enhances excitability (Nitsche and Paulus, 2000).

**Figure 1:**
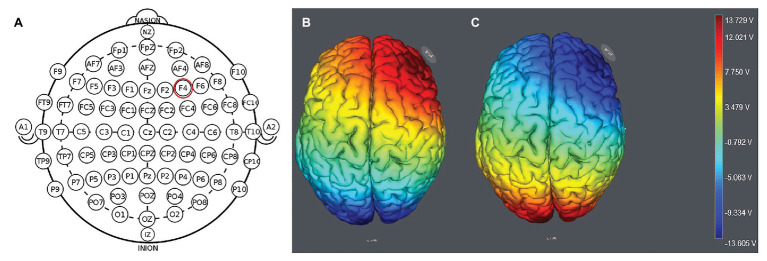
Location of the electrode position **(A)** and the stimulation modes of the two treatments. Anodal stimulation over the right DLFPC **(B)** and cathodal stimulation over the right DLPFC **(C)**.

**Figure 2:**
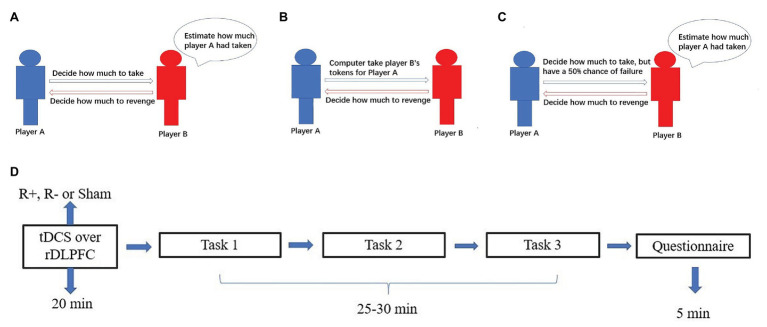
The process for task 1 **(A)**, task 2 **(B)**, task 3 **(C)**, and schematic representation of the experimental design **(D)**. After 20min of stimulation, the participant was asked to complete task 1, task 2, and task 3.

We placed the electrodes on the F4 location (Figure 1). The participants were randomly assigned to one of the three stimulation treatments: anodal stimulation over the right DLPFC; cathodal stimulation over the right DLPFC (Figure 2); and sham stimulation. A constant current of 1.5mA to F4 and Oz was applied for 20min. In the classical protocols, tDCS delivers a low-intensity constant current, varying between 1 and 2mA (Sellaro et al., 2016). Most previous studies used a 1.5mA current (Riva et al., 2012, 2015; Sellaro et al., 2016), and some studies used a 2mA current (Kelley et al., 2013; Sellaro et al., 2016). We chose the 1.5mA current in the present study. Following the standard tDCS protocol, stimulation commenced after a 30-s ramp-up period, and the current was ramped down over the last 30s. For sham stimulation, the current lasted only 30s. This has proven to be reliable because the brief duration of stimulation could hardly modulate cortical excitability, but the participants may feel the initial itching and believe they were receiving stimulation (Gandiga et al., 2006).

### Experimental Task and Procedure

#### Task 1

Task 1 involved three stages. In the first stage, all participants were given 24 tokens and randomly divided into pairs. In the second stage, player A could take player B’s tokens. In the third stage, player B could reduce player A’s tokens by three times the amount player B spends. For example, if player A decided to take 4 tokens from player B in the second stage, then the numbers of player A’s and player B’s tokens were 28 and 20, respectively. In the third stage, if player B decided to spend 6 tokens to reduce player A’s tokens, then the number of player A’s tokens were reduced to 10 and the number of player B’s tokens was 14. At the beginning of task 1, all participants knew all the details of stage 1, stage 2, and stage 3. To measure the participants’ retaliatory behavior, we incorporated the strategy method in which player B in the third stage had to decide on a contingent action for every possible amount taken by player A, which has been proven reliable for measuring participants’ behavior such as trustworthiness (Ashraf et al., 2006; Brandts and Charness, 2011). We can obtain all cases of player B’s revenge behavior when player A takes away 1, 2, 3, …, or 12 tokens. Then players’ tokens were calculated at the end of the experiment. For example, player A decides to take away 4 tokens from player B in the second stage. At the beginning of the third stage, player B did not know the number of tokens taken away by player A. She had to decide for every condition her tokens were taken away by player A. If player B decided to spend 4 tokens to revenge for the condition 4 tokens were taken away by player A and decided to spend 5 tokens to revenge for the condition 5 tokens were taken away, then player B would end up spending 4 tokens to reduce player A’s tokens.

After the third stage, player B was asked to estimate the amount taken away by her partner, and an accurate estimation was rewarded with one extra token (Figure 2A).

#### Task 2

Task 2 also involved three stages. In the first stage, all participants were given 24 tokens and divided into pairs, which was the same as in task 1. In the second stage, player A did not take player B’s tokens by themselves. At some random probability, the computer would take player B’s tokens for player A. In the third stage, player B could reduce player A’s tokens by three times the amount player B spends. Similar to task 1, we also incorporate the strategy method in which player B had to decide on a contingent action for every possible amount taken by the computer (Figure 2B).

#### Task 3

Task 3 also involved three stages. In the first stage, all participants were given 24 tokens and divided into pairs, which was the same as tasks 1 and 2. In the second stage, player A could take player B’s tokens by themselves. In contrast to task 1, player A had a 50% chance of failure. That is, player A had a 50% chance of taking away player B’s tokens successfully and a 50% chance of taking away player B’s tokens unsuccessfully. Similar to task 1 and task 2, we adopted the strategy method. To be more specific, player B had to decide on a contingent action for the conditions in which player A successfully or unsuccessfully took her tokens (Figure 2C).

From task 1 to task 3, we restricted the number of tokens that player A could take to a maximum of 12 tokens. Moreover, we also used the restriction that player A’s tokens could be reduced by player B to zero at most. The tokens are converted into CNY at the end of the experiment. Therefore, the profits were determined by the number of participants’ tokens.

### Experimental Procedure

The experimental software z-Tree was used to present the tasks as well as to automatically calculate participants’ final payoff. The whole experiment was performed in three phases (Figure 2D). In the first phase, the participants received stimulations for 20min (anodal, cathodal, or sham stimulation). In the second phase, the participants completed task 1, task 2 and task 3 according to their roles. However, the participants were randomly assigned as player A or player B.

Moreover, the participants had to pass a control test before entering every task to ensure that they fully understood how the profits were determined. Moreover, when the participants completed task 1, they did not know any of the details regarding task 2 and task 3. Similarly, when the participants took part in task 2, they did not know any of the details regarding task 3. Every participant’s partner was different in task 1, task 2 and task 3. In the third phase, the participants were asked to complete a questionnaire before they finally received their payment. The questionnaire contained questions about their personal information, such as gender, age, income, and consumption expenditure. The participants were informed about how their decisions determined their final payments: every task was played once with each participant randomly paired with another participant, and in the second stage of the experiment, the role each participant played in this game was also randomly assigned by the computer. In addition, the participants were informed of the results from task 1, task 2, and task 3 at the end of the experiment.

## Data Analysis

The critical variables were participants’ revenge behaviors when their tokens were seized in different ways. As we incorporated the strategy method that player B had to decide on a contingent action for every possible amount taken by player A, the average ratio of the individual’s revenge for each token taken away by player A was defined as the average revenge ratio. The average revenge ratio represents the average number of tokens that player B wants to spend for each token taken away by player A. For example, assuming that the revenge was 1 token (“revenge1”) if 1 token was taken away by player A, and the revenge was 2 tokens (“revenge2”) if 2 tokens were taken away by player A, and the revenge was 3 tokens (“revenge3”) if 3 tokens were taken away by player A, etc., then the following was used to calculate the average revenge ratio.

$$\begin{array}{l} Average\;revenge\;ratio=(revenge\frac{1}{1}+revenge\frac{2}{2}+\\ \;\;\;\;\;\;\;\;\;\;\;\;\;\;\;\;\;\;\;\;\;\;\;\;\;\;\;\;\;\;\;\;\;\;\;\;\;\;\;\;\;\;\;\;\;\;\;\;revenge\frac{3}{3}+...+revenge\frac{12}{12})/12. \end{array}$$

Moreover, assuming that the revenge was 0.5 tokens (“revenge1”) if 1 token was taken away by player A, and the revenge was 1 token (“revenge2”) if 2 tokens were taken away by player A, and the revenge was 1.5 tokens (“revenge3”) if 3 tokens were taken away by player A, etc., then the average revenge ratio is 0.5, which means when 6 tokens are taken away by player A, B spends 3 tokens.

We first concentrated on comparing the revenge behaviors of the participants under three conditions in the sham group. The revenge behaviors were not normally distributed, as assessed by the Shapiro-Wilk test. Therefore, nonparametric tests were performed to analyze the data. Because the data for the three kinds of revenge behaviors were nonindependent samples, the Friedman test was applied to analyze the difference in revenge behaviors under three conditions. To test the causal relationship between the activity of the rDLPFC and participants’ revenge behaviors, we conducted the Kruskal-Wallis test to determine if there were differences in the amount between the three kinds of stimulations. When a significant difference was found, *post hoc* analyses were run to identify specific differences. Finally, the Shapiro-Wilk test was also applied to test whether participants’ expectations of their partners’ token-seizing behavior were normally distributed. If not, the Kruskal-Wallis test was conducted to determine if there were differences between the three kinds of stimulations.

All data were statistically evaluated using Stata software. The significance level was set at 0.05 for all analyses. The means and standard errors of the revenge behaviors are shown in Table 1.

**Table 1:** Means and SE of the data for average revenge behaviors under three conditions.

Task	Anodal	Cathodal	Sham	Total
Task 1	0.806 (0.129)	0.320 (0.048)	0.501 (0.093)	0.542 (0.059)
Task 2	0.416 (0.099)	0.208 (0.047)	0.341 (0.089)	0.322 (0.048)
Task 3	0.366 (0.083)	0.103 (0.032)	0.236 (0.078)	0.234 (0.041)

## Results

### Revenge Behavior in the Sham Group

**Figure 3:**
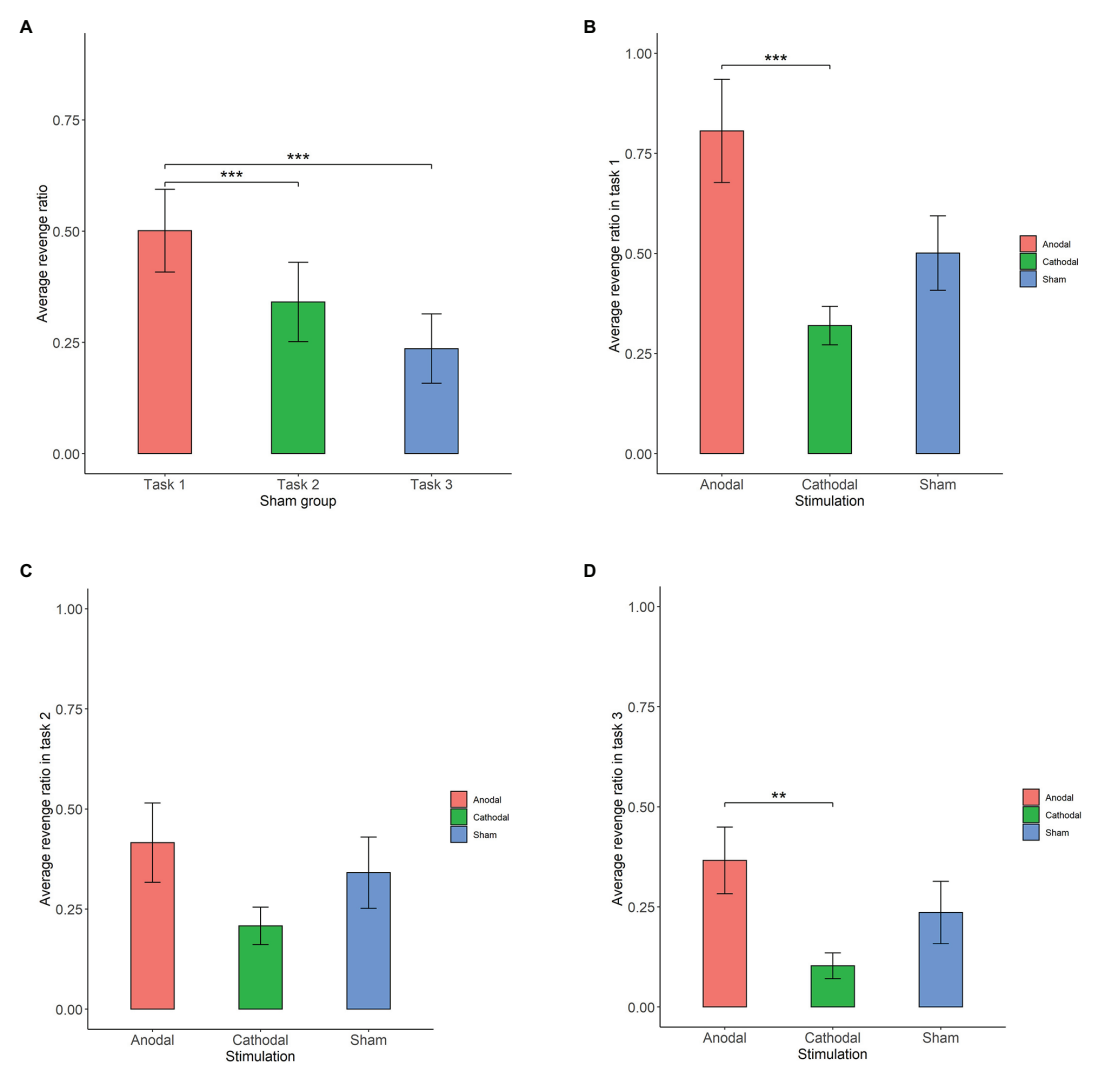
Average revenge ratio in sham groups **(A)**, task 1 **(B)**, task 2 **(C)**, and task 3 **(D)**. Error bar represents standard error. Asterisks indicate significant differences in behavior, ^**^p<0.05, ^***^p<0.01.

First, we examined whether there was any significant difference in participants’ revenge behaviors across the three conditions in the sham group (Figure 3A). The Shapiro-Wilk test showed that the revenge behaviors in the sham group were not normally distributed (*p*<0.01). Based on this, we adopted the Friedman test to analyze the differences in revenge behaviors across the three conditions in the sham groups. The Friedman test showed that there was a significant difference in revenge behaviors across the three conditions ($\chi_{d.f.2}^{2}=130.87$, *p*<0.001). *Post hoc* analyses revealed that participants’ revenge behavior was significantly higher in task 1 than in task 2 (*p*<0.01) and in task 3 (*p*<0.01). However, we did not find a significant difference in participants’ revenge behavior between task 2 and task 3 (*p*>0.1). These results indicated that the participants’ revenge behavior depended on the conditions in which their tokens were taken away.

### Revenge Behavior in Task 1: The Stimulation Effect

The Shapiro-Wilk test showed that the revenge behavior in task 1 was not normally distributed (*p*<0.01). To test the stimulation effect, we adopted the Kruskal-Wallis test to determine whether there was a difference in the amount offered for retaliation among the three stimulation conditions. The Kruskal-Wallis test revealed that there was a significant difference in revenge behavior across the three stimulation conditions ($\chi_{d.f.2}^{2}=8.609$, *p*=0.014). *Post hoc* analysis (H test) showed that revenge behavior was significantly increased after receiving anodal stimulation compared with cathodal stimulation (FDR-adjusted (BH), *p*<0.01). Although revenge behavior was increased after receiving anodal stimulation compared with sham stimulation, the difference was not significant (FDR-adjusted *p*=0.069). Moreover, the revenge behavior after receiving cathodal stimulation was lower than that after receiving sham stimulation, but the difference was not significant (FDR-adjusted *p*>0.1). This finding indicated that anodal stimulation in the right DLPFC made participants more vengeful when their tokens were taken away. Moreover, this finding also indicated that cathodal stimulation in the right DLPFC had no such effect when participants’ tokens were taken away by others (Figure 3B).

**Figure 4:**
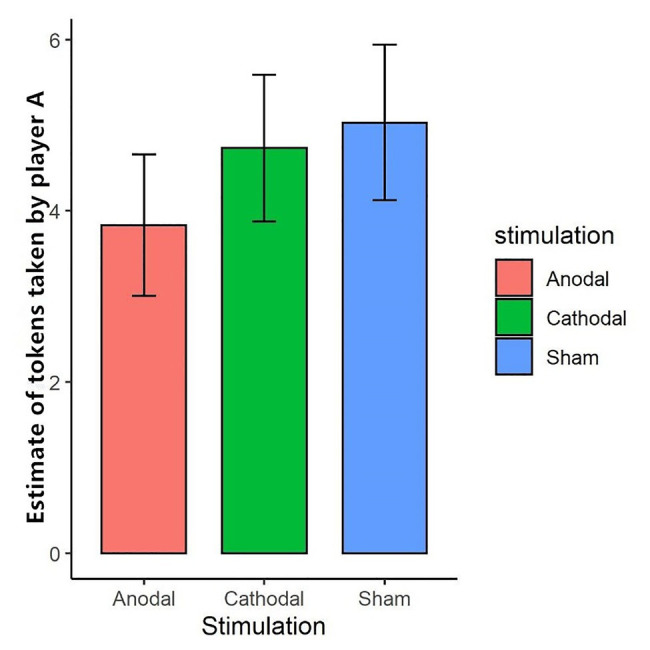
Estimate of tokens taken by player A in task 1 under three stimulations. After the third stage, player B was asked to estimate the amount taken away by her partner. “Expected occupation” represents the amount player B estimated. Error bar represents standard error.

We further tested the effect of tDCS on participants’ expectations of their partner’s token-seizing behavior. The Shapiro-Wilk test showed that expected token-seizing behavior in task 1 was not normally distributed (*p*<0.01). To test the stimulation effect, we adopted the Kruskal-Wallis test to determine whether there was a difference in expected token-seizing behavior among the three stimulation conditions. The results showed that the difference was not significant (*p*>0.1). This finding indicated that anodal and cathodal stimulation in the right DLPFC did not change the participants’ expectations of their partner’s token-seizing behavior while enhancing activity in the right DLPFC made the participants more vengeful (Figure 4).

### Revenge Behavior in Task 2: The Stimulation Effect

The Shapiro-Wilk test also showed that the revenge behavior in task 2 was not normally distributed (*p*<0.01). The Kruskal-Wallis test showed that the different types of stimulation did not significantly affect revenge behavior in task 2 ($\chi_{d.f.2}^{2}=1.515$, *p*>0.1). This finding indicated that anodal and cathodal stimulation in the right DLPFC did not make participants more vengeful when their tokens were randomly taken away by computer (Figure 3C).

### Revenge Behavior in Task 3: The Stimulation Effect

The Shapiro-Wilk test also showed that revenge behavior across conditions was not normally distributed (*p*<0.01). The Kruskal-Wallis test showed that the different types of stimulation significantly affected revenge behavior in task 3 ($\chi_{d.f.2}^{2}=6.974$, *p*=0.031). *Post hoc* analysis revealed that revenge behavior was higher after receiving anodal stimulation than after receiving cathodal stimulation (FDR-adjusted *p*=0.021). Revenge behavior was higher after receiving anodal stimulation than after receiving sham stimulation, but the difference was not significant (FDR-adjusted *p*=0.056). In addition, revenge behavior after receiving cathodal stimulation was not significantly different from that after receiving sham stimulation (FDR-adjusted *p*>0.1). This finding indicated that anodal stimulation in the right DLPFC made participants more vengeful when other participants simply intended to take away their tokens (Figure 3D).

### Order Effect

To further test whether there was an order effect, we added three treatments of behavioral experiments. We recruited 124 new participants to further test whether there was an order effect. In the first experiments, participants completed task 1, task 2, and task 3 in turn (*n*=40, 24 females). In the second experiment, participants completed task 2, task 1, and task 3 in turn (*n*=42, 24 females). In the third experiment, participants completed task 3, task 2 and task 1 in turn (*n*=42, 24 females). Every experiment lasted approximately 40min; on average, participants received a payment of approximately 35.60 CNY (approximately 4.81 US dollars). The Kruskal-Wallis test revealed that revenge behavior in task 2 was not influenced by the order ($\chi_{d.f.2}^{2}=1.115$, *p*=0.573). In addition, The Kruskal-Wallis test also revealed that revenge behavior in task 3 was not influenced by the order ($\chi_{d.f.2}^{2}=0.964$, *p*=0.617).

## Discussion

Although retaliation is universal in our daily lives, its costs are high, and the consequences are severe. As retaliation is a complex process, revenge remains somewhat of a puzzle among human behaviors, and a series of previous studies from different fields have discussed the issues of retaliation and retribution. Research has shown that many brain regions, such as the ACC, IFG, ventrolateral prefrontal cortex, ventromedial prefrontal cortex and DLPFC, have been implicated in revenge behavior (Kerns et al., 2004; Egner and Hirsch, 2005; Sanfey, 2007; Tabibnia et al., 2008; Brüne et al., 2013; Ricciardi et al., 2013). However, the previous findings are mixed, and it worth noting that revenge behavior under conditions in which wealth was not taken but the loss of wealth was anticipated has seldom been examined.

The present study with tDCS complements these studies by providing a causal relationship between revenge behavior and the activities of the right DLPFC. In addition, we explored revenge behavior under three different conditions. To be more specific, we adapted the moonlighting game (Abbink et al., 2000) and designed a new paradigm. Based on this procedure, we examined participants’ revenge behavior in the following three conditions: participants’ wealth was directly taken away by others, participants’ wealth was taken away and given to others by a computer, and seizure of participants’ wealth did not occur, but the loss of wealth was anticipated. The findings of the present investigation provide new evidence and reinforce conclusions from previous neuroimaging and brain stimulation studies (Knoch et al., 2006; Strang et al., 2015; Müller-Leinß et al., 2018; Maier et al., 2019). Our findings also show the possibility and feasibility of combining brain stimulation with social-psychological manipulation to reduce revenge behavior.

According to the behavioral data from the participants in the sham group across the three conditions, we found that participants’ revenge behavior depended on how wealth was taken away. That is, when wealth was directly taken away by others, the revenge behavior was significantly higher than that when wealth was taken away by a computer. Similarly, the revenge behavior when wealth was directly taken away by others was also significantly higher than that when wealth was not lost but the loss of wealth was anticipated. However, there was no significant difference between the conditions in which wealth was taken away and given to others by a computer and when the loss of wealth did not occur but was anticipated. The results indicated that participants cared about the manner in which their wealth was taken away, which was consistent with a previous study (Ruff et al., 2013).

Based on the above behavioral results, we further explored the neural evidence regarding revenge behavior when participants’ wealth was taken away by others. According to the data in task 1, when activity in the right DLPFC was enhanced, the participants engaged in a greater level of revenge than when activity in the right DLPFC was not changed in the sham group. Moreover, the participants also engaged in more revenge following anodal stimulation than following sham stimulation, but the difference was not significant. In addition, the difference between cathodal stimulation and sham stimulation was not significant. This seemed to indicate that the function of the right DLPFC is to override basic human impulses related to self-interest, which was consistent with previous studies (Knoch et al., 2006).

As mentioned above, the previous findings of the relationship between the right DLPFC and retaliation are puzzling. Some findings have revealed that disruptions in the right DLPFC decreased participants’ possibility of rejecting unfair offers, which indicated that participants engaged in less revenge (Knoch et al., 2006; Strang et al., 2015). Some findings using noninvasive brain stimulation, such as RTMS and cTBS, have argued that inhibiting the right DLPFC increased retaliation (Müller-Leinß et al., 2018; Maier et al., 2019). In the latter set of studies, the experimental settings did not distinguish the wish to maximize one’s own gain and the desire to retaliate. That is, participants in the experiment could maximize their monetary gain by retaliating against their opponents (Müller-Leinß et al., 2018). However, in our present study, there was a trade-off between one’s own gain and retaliation, which was in line with the former set of studies (Knoch et al., 2006; Strang et al., 2015). The results revealed that enhancing activity in the DLPFC increased participants’ revenge behavior, which was similar to the former set of studies (Knoch et al., 2006; Strang et al., 2015). Our results seemed to be at odds with the latter set of studies (Müller-Leinß et al., 2018; Maier et al., 2019). However, our results showed that participants who engaged in more revenge gained less following enhancement of activity in the DLPFC, and the latter set of studies also showed that participants who engaged in more revenge gained less following inhibition in the right DLPFC (Müller-Leinß et al., 2018; Maier et al., 2019). From the point of view that the function of the right DLPFC is to override self-interest, our present findings are also consistent with the latter set of studies (Müller-Leinß et al., 2018; Maier et al., 2019).

Interestingly, although enhancing activity in the right DLPFC changed participants’ revenge behavior, tDCS did not change participants’ anticipation of the amount taken away by others. We measured participants’ expectations of their partners’ token-seizing behavior. The Kruskal-Wallis test indicated no significant difference in the expected loss of tokens across the treatments. Thus, enhancing activity in the right DLPFC changed revenge behavior but did not affect the anticipation of losing tokens. This finding is similar to previous studies (Knoch et al., 2006; Ruff et al., 2013) in which participants’ beliefs, such as fairness and perceived anger, were not changed by brain stimulation.

To further explore the psychological mechanisms that may have contributed to the tDCS effect, attention was given to the way participants’ wealth was taken away. We measured participants’ revenge behavior when their wealth was not directly taken away by their opponents but randomly taken away and given to their opponents by a computer. According to the data obtained in task 2, no significant difference in revenge was found across the three stimulation conditions. The results revealed that altering activity in the right DLPFC did not change participants’ revenge behavior when their tokens were taken away and given to their opponents by a computer, which is consistent with the previous study (Knoch et al., 2006). This finding was also similar to Ruff’s study (Ruff et al., 2013) in which participants’ behavior was less affected when opponents were not humans but a computer.

Furthermore, we analyzed participants’ retaliation when the loss of wealth was anticipated. We measured participants’ revenge behavior in the condition in which the tokens were not taken but the loss of tokens was anticipated. According to the data obtained in task 3, participants’ retaliation levels were higher when activity in the right DLPFC was enhanced than when activity in the right DLPFC was attenuated. However, participants’ retaliation levels were not significantly higher in the anodal stimulation group than in the sham stimulation group. This finding indicated that the DLPFC is crucial in revenge behavior related to the motivation for invasion.

Thus, together with the results above, enhancing activity in the right DLPFC changed revenge behavior when wealth was taken by a person but did not affect revenge behavior when wealth was taken away and given to others by a computer. Revenge behavior is thought to have played an important role in the evolution of human behavior and survival (Seymour et al., 2007; Rilling and Sanfey, 2011; McCullough et al., 2013; Akin and Akin, 2016; Nowak et al., 2016). Our results revealed that the right DLPFC is crucial in revenge behavior related to the motivation of invasion.

In the experimental procedure, because task 1 is always before task 2 and task 3, there may be an order effect. It is certain that task 1 is not influenced because participants did not know any details of task 2 and task 3 before they completed the task1. However, task 2 and task 3 may be influenced by the order effect. To reduce the order effect, we arranged that participants did not know the results of task 1 when they made decisions in task 2 and task 3. Moreover, participants did not know the results of task 2 when they made decisions in task 3. To be more specific, all the tasks’ profits were shown to participants at the end of the experiment. In addition, participants were repaired in pairs in task 2 and task 3, respectively. Thus, every participant’s partner was different in task 1, task 2, and task 3. Certainly, that is not enough to completely avoid the order effect. To further test whether there was an order effect, we added three treatments of behavioral experiments. The Kruskal-Wallis test revealed that revenge behavior in task 2 and task 3 was not influenced by the order. In classical protocols, tDCS delivers a low-intensity constant current, varying between 1 and 2mA (Sellaro et al., 2016). When the current is delivered for a sufficient period of time (i.e., at least 9–10min), the effect can remain for longer than 1h after stimulation (Nitsche and Paulus, 2000, 2001; Nitsche et al., 2003; Sellaro et al., 2016). Moreover, long-lasting excitability elevations can be induced in the human motor cortex by weak continuous transcranial direct current stimulation. To detect current-driven changes of excitability, motor-evoked potentials (MEP) were usually recorded. In a previous study (Nitsche and Paulus, 2000), 5- and 7-min tDCS resulted in after-effects lasting for no longer than 5min, tDCS from 9 to 13min resulted in elevations of MEP amplitudes from 30 (9-min tDCS) to 90 (13-min tDCS) minutes. For the longer tDCS, MEP show no linear decrease for a relatively long time but are fairly stable before returning to baseline levels. In our present study, the current was delivered for 20min, and the tasks lasted about 25–30min in total. Therefore, the effect may not decrease over the 3 tasks. However, further research should pay attention to counterbalancing task 1, task 2, and task 3.

Although our findings revealed that altering excitability in the DLPFC changed participants’ revenge behavior when their wealth was directly taken away by others, the present study has some relevant limitations. First, the neural circuitry underlying the decision-making process of revenge behavior cannot be demonstrated by a single experiment. Second, the involvement of other prefrontal areas, such as the ventromedial and anterior prefrontal cortex, was not examined. Third, a between-subject design may have had the advantage of comparing revenge behaviors under different conditions. Fourth, the second electrode is positioned at Oz because it is off the head and thus less likely to affect a response in the brain. Moreover, previous studies have shown that the occipital cortex lobe is not related to revenge behavior (Brüne et al., 2013; Maier et al., 2019). However, future studies should pay attention to side effects. Futhermore, the range of subjects’ ages was narrow. The average age of females was 20.22, ranging from 18 to 25. The average age of males was 20.30, ranging from 18 to 27. However, our studies are in line with previous studies. Twenty-nine healthy subjects were enrolled in Brüne’s study, and the average age was 27.9, ranging from 21 to 37years. They found that revenge behavior is associated with the activation of the DLPFC (Brüne et al., 2013). Sixty-seven subjects were recruited in Maier’s study (mean age was 34.4). Considering age and sex, no significant differences in revenge behavior were observed (Maier et al., 2019). In a rTMS study, 46 healthy subjects (25 women) were recruited. The mean age of all the subjects is 24.59. The results indicated that subjects showed increased revenge behavior after the inhibition of the right DLFPC in the UG and DG games. Our study is in line with this result regarding whether subjects maximize self-interest in revenge behavior (Müller-Leinß et al., 2018).

In addition, future studies may focus on the examination of other brain regions and the neural circuitry of the DLPFC. Moreover, revenge behaviors when participants’ wealth is taken away in different ways could also be studied using a between-subject design. Furthermore, future studies may adopt neuroimaging measures and rTMS to study the neural changes associated with neuroimaging measures.

## Data Availability Statement

The raw data supporting the conclusions of this article will be made available by the authors, without undue reservation.

## Ethics Statement

The studies involving human participants were reviewed and approved by Zhejiang University of Finance and Economics Ethics Committee. The patients/participants provided their written informed consent to participate in this study.

## Author Contributions

WZ and YT: conceptualization. WZ, JL, and HY: methodology. WZ, YL, and HY: validation. WZ and JL: writing – review and editing. All authors have read and agreed to the published version of the manuscript.

### Conflict of Interest

The authors declare that the research was conducted in the absence of any commercial or financial relationships that could be construed as a potential conflict of interest.
